# Changes in acute and trauma hand surgery in the first Covid-19 lockdown in a German trauma center: a retrospective analysis of 338 cases

**DOI:** 10.1007/s00402-021-04319-8

**Published:** 2022-02-18

**Authors:** Marie-Luise Klietz, Matthias M. Aitzetmüller, Johannes Glasbrenner, Michael J. Raschke, Martin F. Langer, Simon Oeckenpöhler

**Affiliations:** 1 Section for Plastic and Reconstructive Surgery, Department for Traumatology, University Hospital Münster, Münster, Germany; 2 Department of Trauma, Hand and Reconstructive Surgery, University Hospital Münster, Albert-Schweitzer-Campus 1, Gebäude W1, 48149 Münster, Germany

**Keywords:** Hand surgery, Covid-19, Corona, Hand trauma, Public health, Epidemiology

## Abstract

**Introduction:**

Although Covid-19 and especially lockdown periods have affected our everyday live, its impact on hand traumatology is under investigated.

**Materials and methods:**

We retrospectively analyzed all patients presenting at a FESSH accredited HTRC and level 1 trauma center in Germany during the Covid-19 lockdown period and an equivalent timeframe in 2019 regarding incidence of hand trauma, injury mechanism, type of injury and hand surgeries.

**Results:**

338 patients presented at our department with acute hand injuries. A significant reduction of work-related accidents was found during lockdown contrary to an increase of do-it-yourself related trauma. Although the incidence of hand trauma decreased during lockdown by 18%, the rate of hand surgery increased in absolute and relative numbers.

**Conclusions:**

Although Covid-19 has negatively impacting elective and semi-elective surgeries, acute hand surgery has gained in importance represented by a shift from work related to do-it-yourself trauma and an increased rate of surgical treatment.

**Level of evidence:**

IV (therapeutic).

## Introduction

After the first European Covid-19 infection was detected on January 24th in France [1], it spread all over Europe and led to a nation-wide lockdown in Germany. In the period between march 18th and May 14th it significantly influenced every day’s life [2]. Nationwide short-time work was introduced, leading to a granted income of 60% (or 67% for those with children) compensating the cut in working hours. Thereby, average time at home rapidly increased, by simultaneously reducing professional workload. While hotel and restaurant industry consequently were closed, construction markets were fully opened offering novel do-it-yourself (DIY) possibilities. In combination with enhanced spare time these circumstances were strengthening and awakening craftsmanship abilities of nonprofessionals.

Simultaneously hospitals intended to reduce the number of elective surgeries—especially those with possible need for postoperative intensive care, in order to provide ventilation units for Covid-19 patients. While it has been shown, that the total number of medical emergencies has dramatically decreased during the lockdown period [3, 4], its impact on hand injuries is broadly under investigated.

The Covid-19 pandemic has significantly changed medicine and clinical routine. However, published articles mostly represent case reports, experiences or opinion papers [5–8]. Only few clinical analyses investigating these changes are currently available but are urgently needed to prepare on upcoming periods of lockdown and possible future pandemics.

The goal of the present study was to retrospectively analyze all patients presenting at a FESSH accredited HTRC and level 1 trauma center in Germany during the lockdown period (March 18th until May 14th 2020) and to compare them to the equivalent timeframe in 2019 regarding trauma mechanisms and patient related factors.

It was hypothesized that the incidence of emergency hand surgeries was hardly affected by the lockdown period, with a decrease of working accident and a simultaneous increase of DIY and household accidents.

## Methods

A retrospective clinical trial was conducted over a period of 2 years. The study was carried out at a FESSH accredited HTRC and level 1 trauma center. According to the German Society of Traumatology, the University Hospital is the only level 1 trauma center (“überregionales Trauamzentrum”) within an area of about 300 k inhabitants and a radius of 60 km. The ethical review board of our university approved the study (2020-397-f-S), which was performed in accordance with the Declaration of Helsinki and the guidelines of Good Scientific Practice, as supported by the Head of the Institute.

### Data collection

**Fig. 1:**
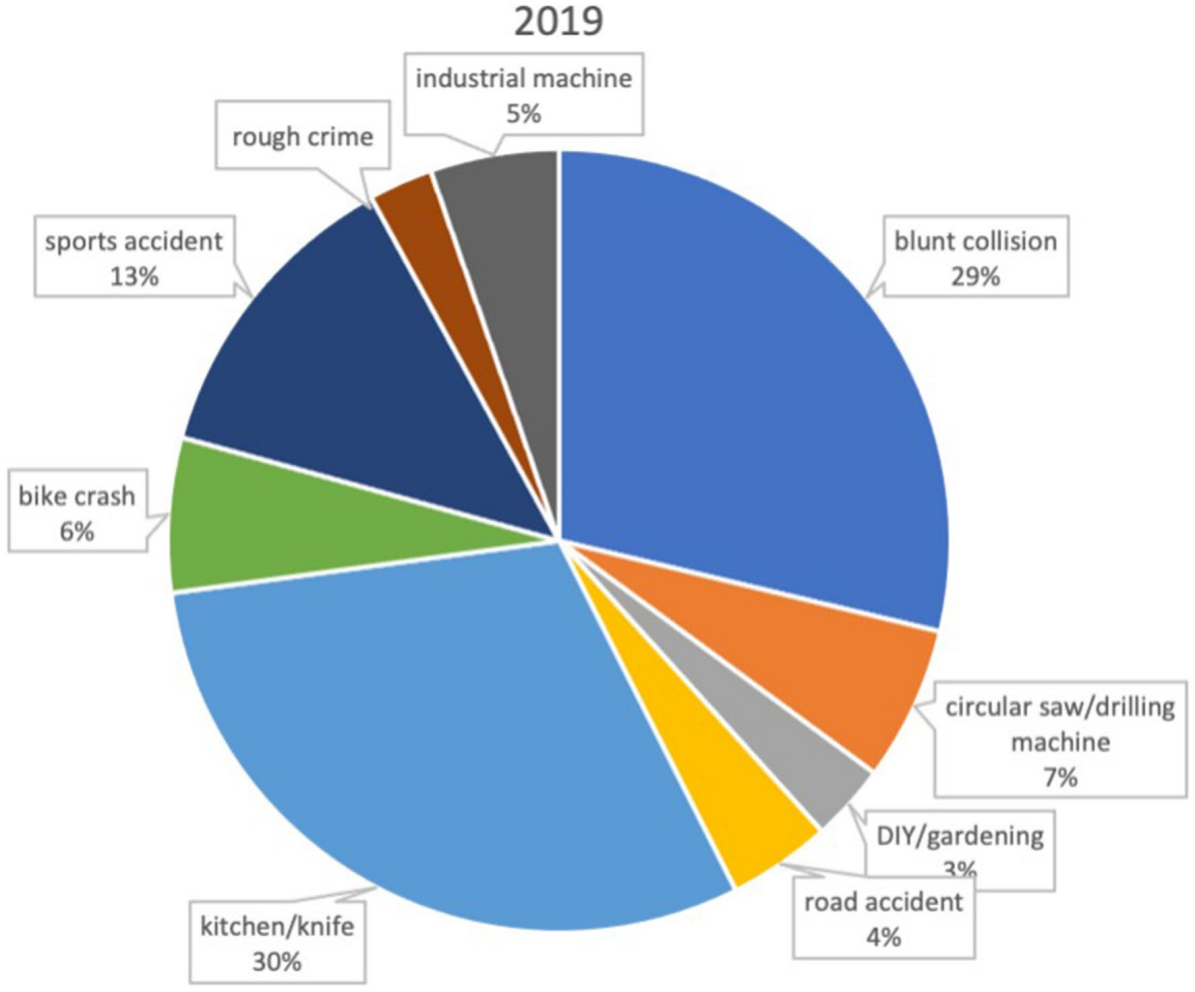
Distribution of trauma mechanism during the nonlockdown period in 2019

**Fig. 2:**
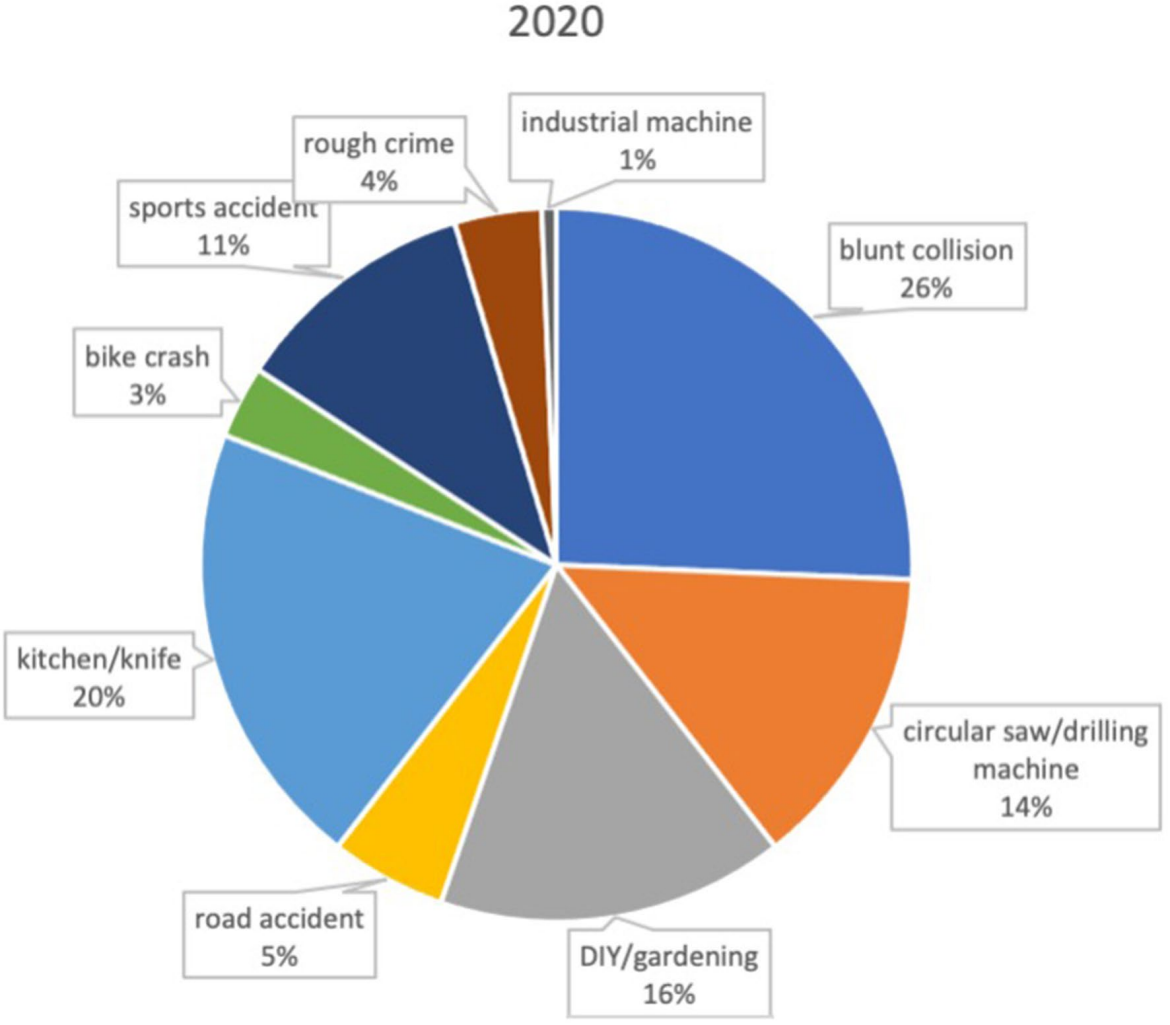
Distribution of trauma mechanism during the first 2020 COVID-19 lockdown period

All patients presenting at our department with an injury of the hand (distal to the radiocarpal joint) within lockdown period (between March 18th and May 14th 2020) were included. Patients presenting within 18th of March and 14th of May 2019 served as control group (Figs. 1, 2).

Anonymized patient data was collected including gender, age and date of injury. Additionally, mechanism of injury, injured structures (isolated soft tissue defect, partial amputation, total amputation, fracture, bruise) as well as the number of affected fingers were evaluated. It was distinguished whether the injury was caused by a private or occupational accident and the trauma mechanism was categorized within the following categories: blunt collision, circular saw/drilling machine, DIY or gardening, road accident, kitchen/knife, bike crash, sports accident, rough crime or industrial machine.

If a surgical intervention was needed, the procedure was carried out by a senior physician of our clinic with assistance of one resident. Duration of surgery and possible additional measures or revision surgeries were documented.

### Statistical analysis

Statistical analysis was carried out using Microsoft Excel (Version 16.45, Microsoft, Albuquerque, New Mexico, USA). All values are given as mean ± standard deviation (SD).

For comparison between groups a two tailed Chi-square test was used. In addition, a two tailed *T* test was used for comparison of qualitative data. A *p* value of 0.05 or lower was considered as statistically significant.

## Results

### Patient demography

In total 338 patients presented at our department within the two defined periods. Of those 186 presented in 2019 and 152 during lockdown in 2020 indicating no significant difference within patient demography between 2019 and 2020 (*p* = 0.59). Mean age was 35 (± 19) with 109 (32.2%) being female and 229 (67.8%) being male.

No significant difference (*p* = 0.33) was found in age distribution, with an average age of 34 (± 19) years in 2019 and 36 (± 19) years in 2020. 59 female (31.7%) and 127 male (68.3%) patients were included in 2019, and 50 patients (32.9%) were female and 102 (67.1%) were male in 2020.

### Trauma mechanism

Categorization was performed based on insurance in work related accidents (covered by the employers’ liability insurance association) or private accidents. While 64 injuries (34%) were work related in 2019, in 2020 only 26 (17.1%) can be seen as working accidents, indicating a significant decrease of 51.7% (*p* = 0.02*).

Trauma mechanism was evaluated in all patients and is listed in Table 1. During lockdown period a significantly enhanced incidence of trauma caused by saws (6.5% in 2019 vs 13.8% in 2020, *p* = 0.025*) and other DIY occupations (3.2% vs 15.8%, *p* < 0.001) was found. Simultaneously the incidence of hand trauma caused by cooking (30.6% vs 20.4%, *p* = 0.03) or during industrial work significantly decreased (5.4% vs 0.6%, *p* < 0.001) (Figs. 1, 2).

**Table 1:** Trauma mechanisms 2019 vs 2020 (absolute and relative)

Trauma mechanism	2019 (*n* = 186)	2020 (*n* = 152)
Blunt collision	54 (29.0%)	39 (25.6%)
Circular saw/drilling machine	12 (6.5%)	21 (13.8%)
DIY/gardening	6 (3.2%)	24 (15.8%)
Road accident	8 (4.3%)	8 (5.3%)
Kitchen/knife	57 (30.6%)	31 (20.4%)
Bike crash	12 (6.5%)	5 (3.3%)
Sports accident	24 (12.9%)	17 (11.2%)
Rough crime	5 (2.7%)	6 (3.9%)
Industrial machine	10 (5.4%)	1 (0.6%)

### Injury characteristics

Traumata were classified according to injury pattern (Table 2), yielding no significant difference between lockdown a nonlockdown period.

**Table 2:** Injury patterns 2019 vs 2020 (absolute and relative)

Injury pattern	2019 (*n* = 186)	2020 (*n* = 152)
Amputation	5 (2.7%)	5 (3.3%)
Partial amputation	8 (4.3%)	7 (4.6%)
Fracture	12 (6.5%)	27 (17.7%)
Bruise	39 (21.0%)	27 (17.8%)
Soft-tissue defect	77 (41.4%)	68 (44.7%)
Abrasion	1 (0.5%)	5 (3.3%)
Tendon injury	24 (12.9%)	12 (7.9%)

**Fig. 3:**
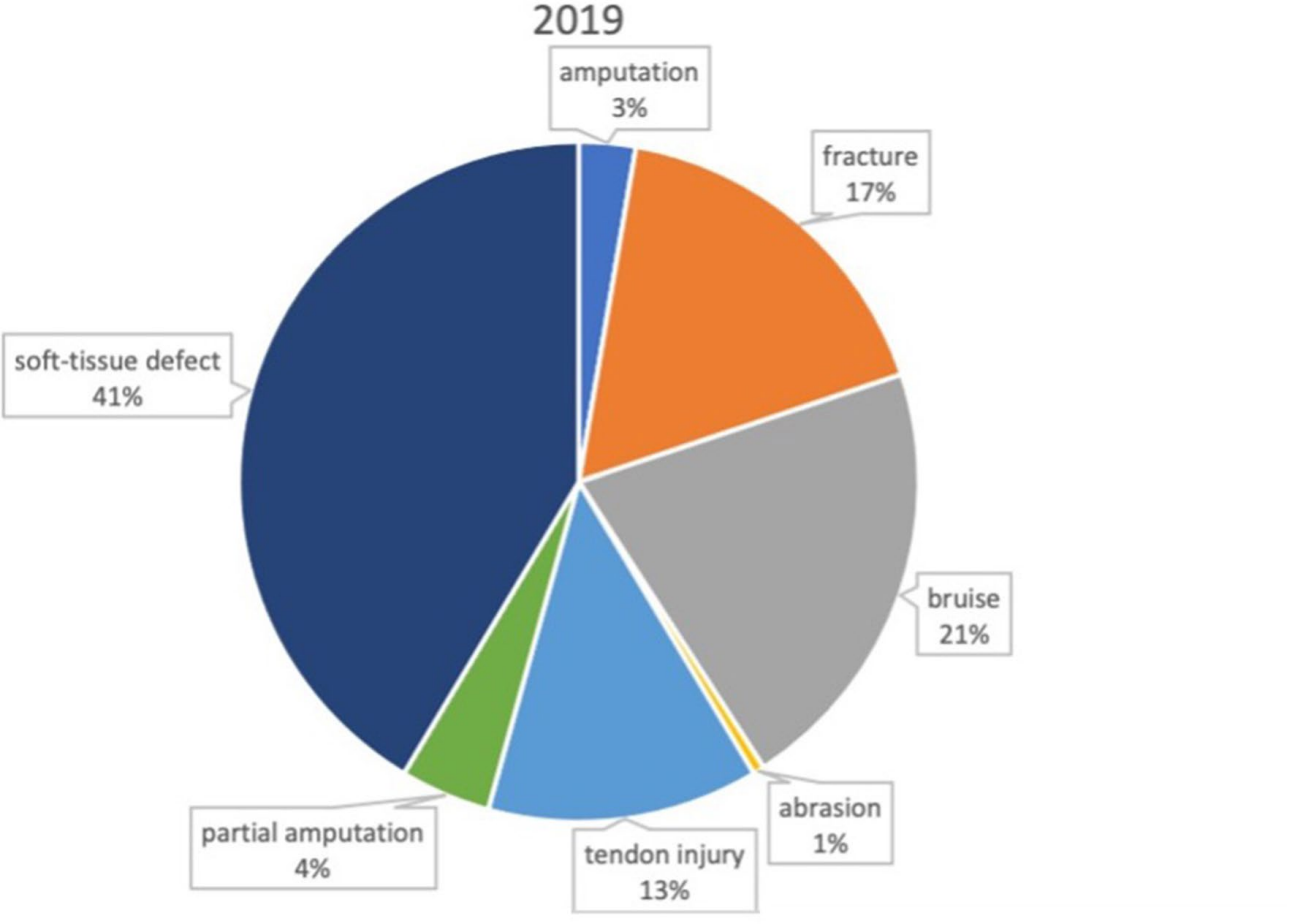
Injury characteristics the nonlockdown period in 2019

**Fig. 4:**
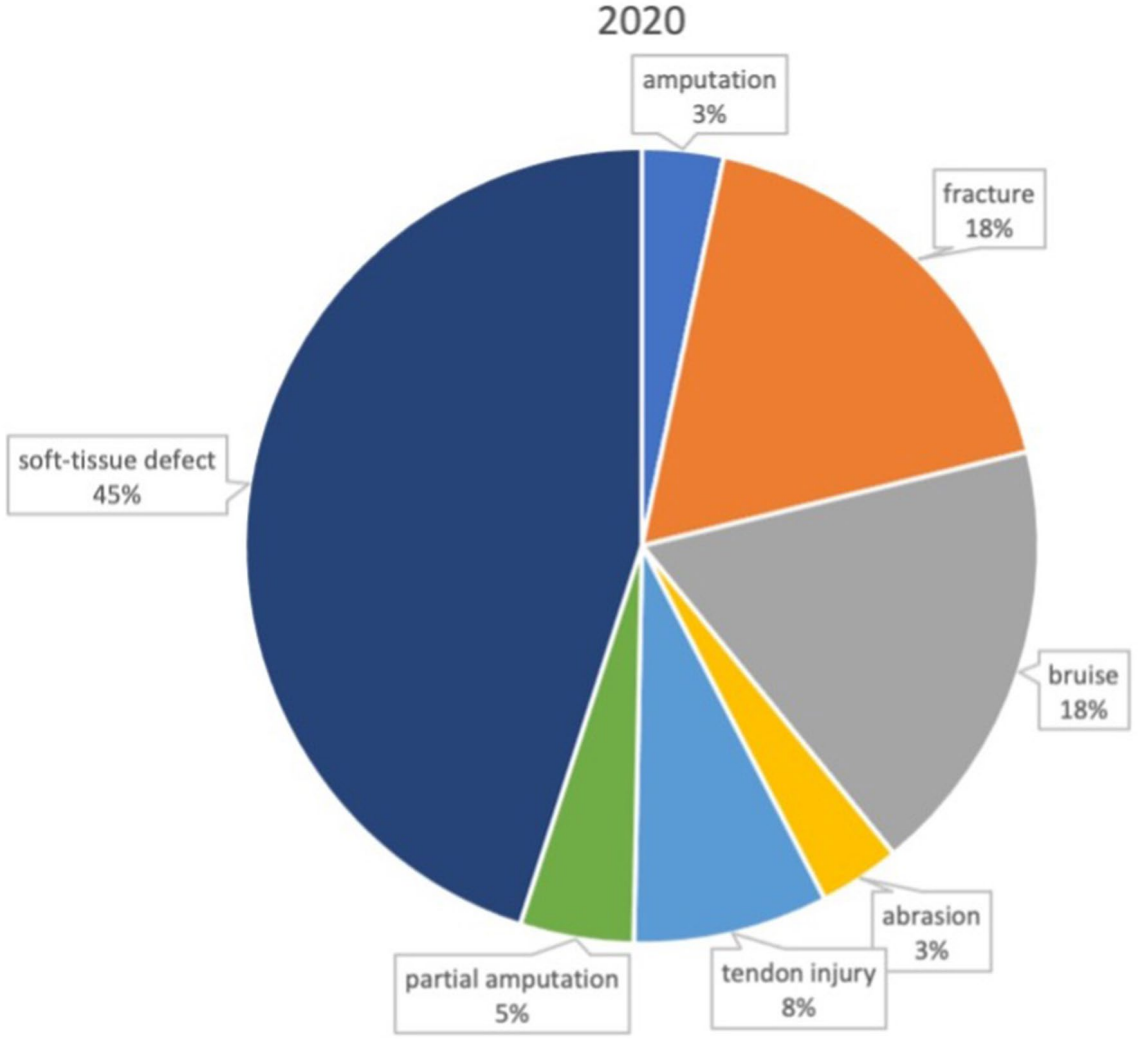
Injury characteristics during the first 2020 COVID-19 lockdown

In the 2019 period a surgical intervention was carried out in 60 Patients, whereas in the 2020 period 67 patients were treated by at least one surgical intervention, highlighting a significant increase in surgery rate (34.2% vs 46.1%, *p* = 0.025). Mean duration of surgery was 68 (± 47) minutes in 2020 compared to 67 (± 83) minutes in 2019 indicating no significant difference between lockdown and nonlockdown period (Figs. 3, 4).

### Case presentations of severe hand injuries occurred during the lockdown

#### Case 1

**Fig. 5:**
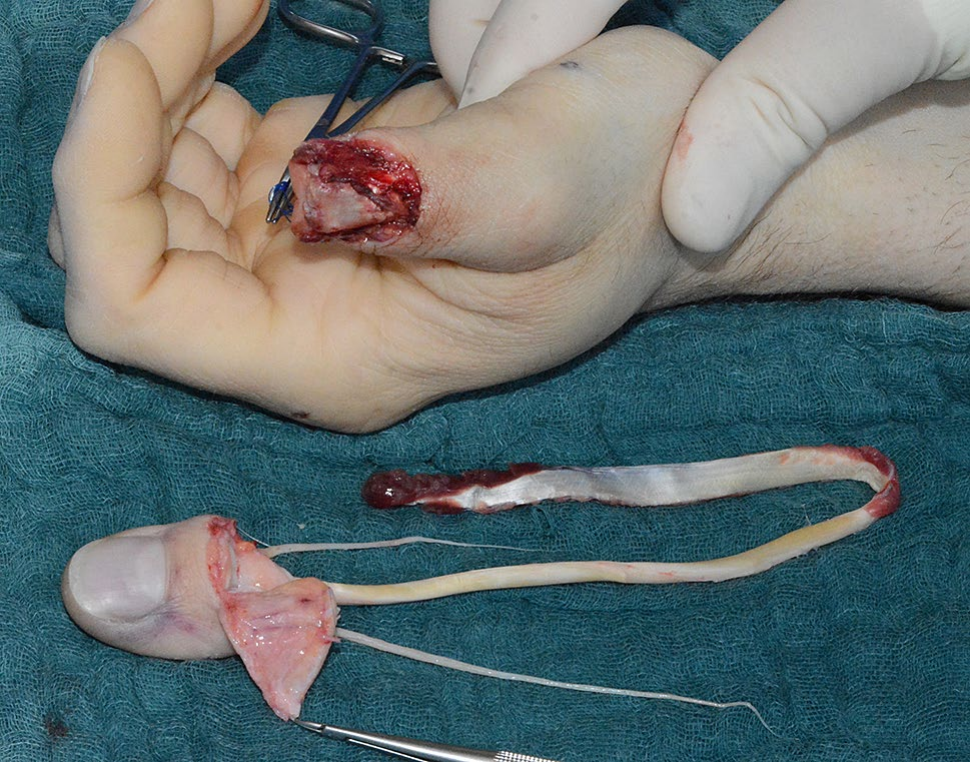
Case Presentation 1: De Marchetti grade III avulsion of distal thumb phalanx

**Fig. 6:**
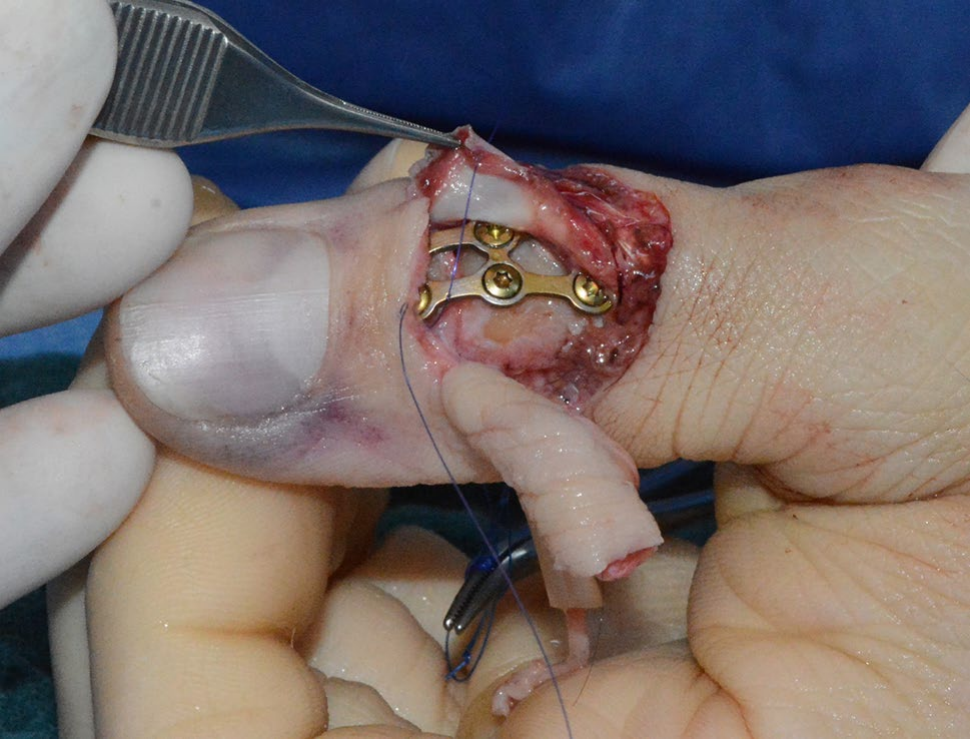
Case Presentation 1: Primary arthrodesis of IP joint

**Fig. 7:**
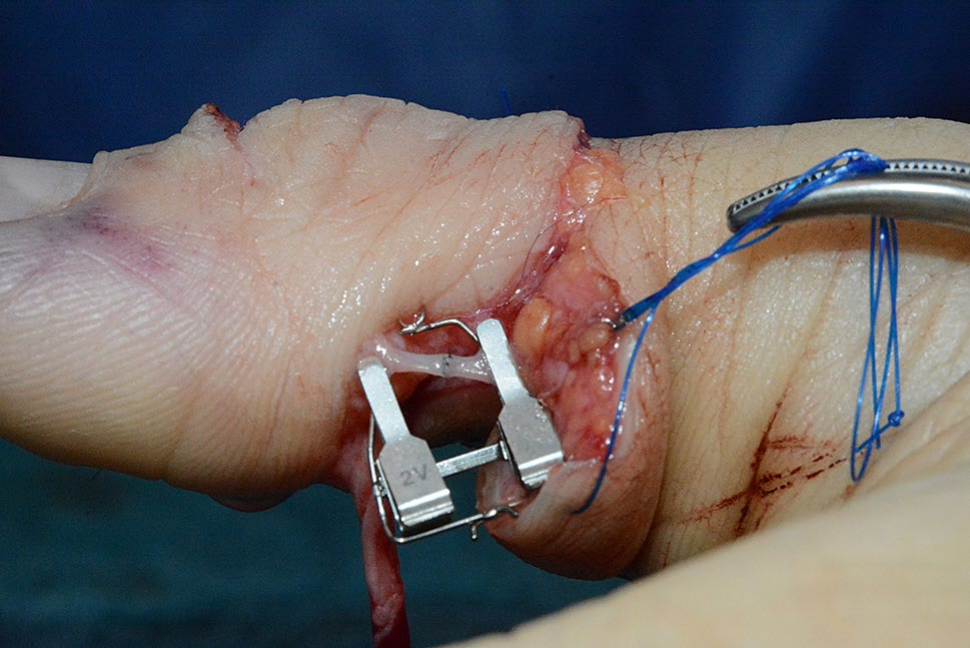
Case Presentation 1: Microvascular anastomosis of A1

**Fig. 8:**
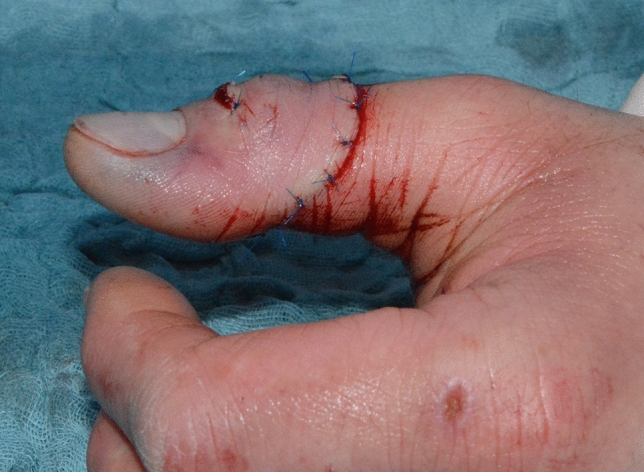
Case Presentation 1: End of surgery result

**Fig. 9:**
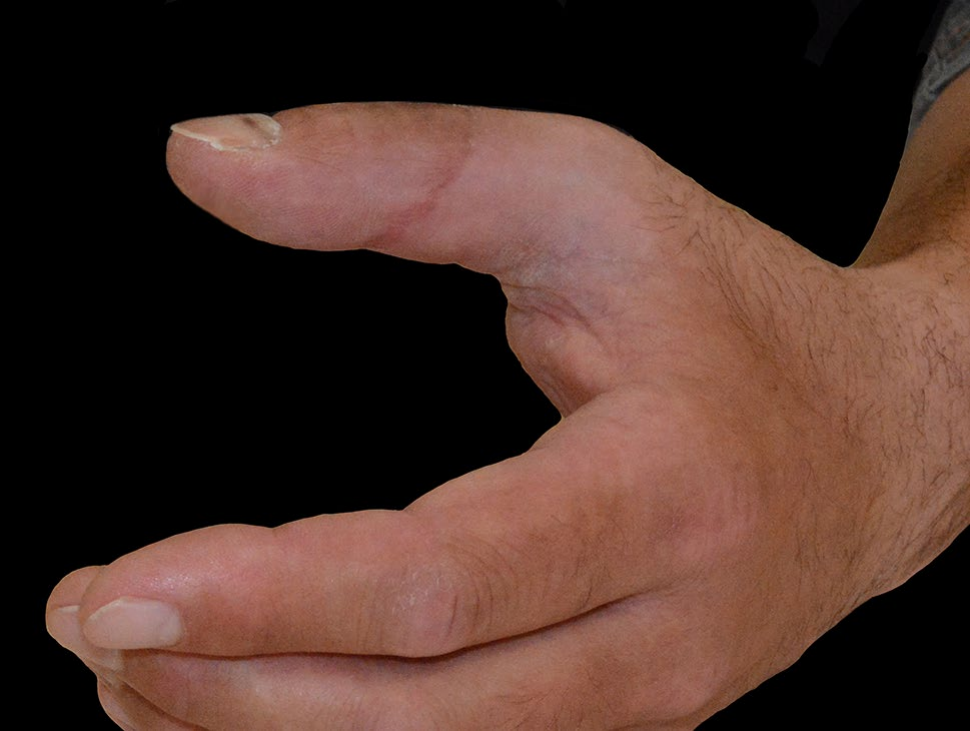
Case Presentation 1: Replantation result 6 months later

Mr. P., a craftsman, presented at our department after working at a drilling machine. Because being afraid of dermal exposure to Covid-19 virus, he was wearing gloves during his work. Thereby the glove became stuck in the drilling machine leading to a complete avulsion of the distal phalanx and the FPL tendon (Marchetti grade III) (Figs. 5, 6, 7, 8, 9).

Immediate replantation in combination with an arthrodesis of the interphalangeal joint was performed. Postoperatively we found a satisfying result with a fully vascularized distal phalanx.

#### Case 2

**Fig. 10:**
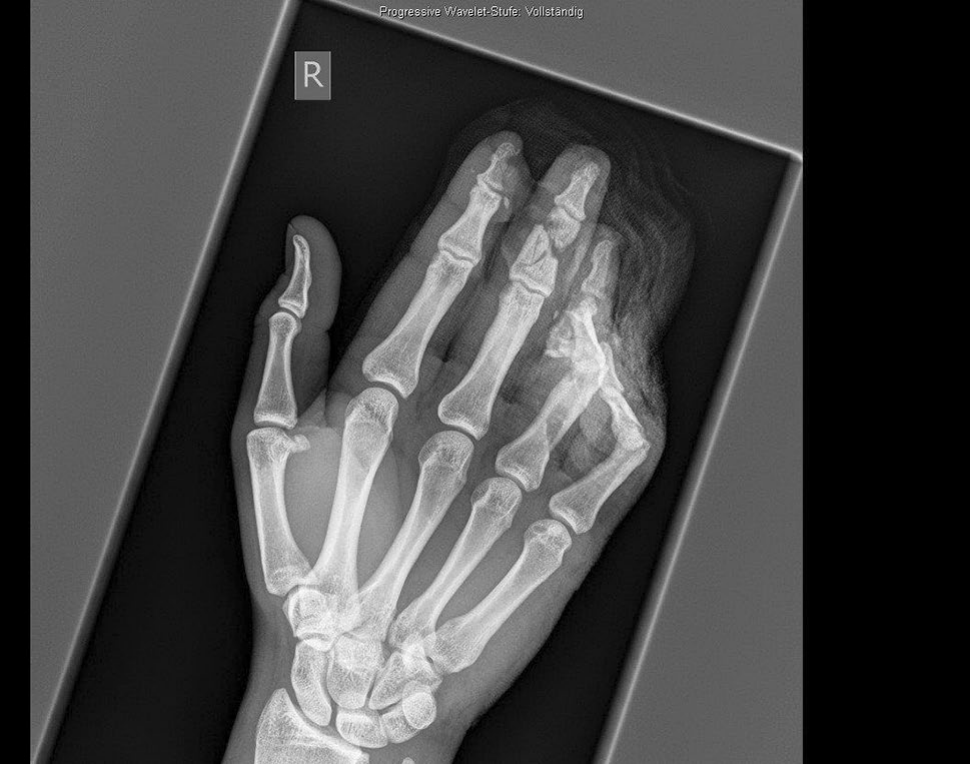
Case Presentation 2: X-ray after circular saw injury

**Fig. 11:**
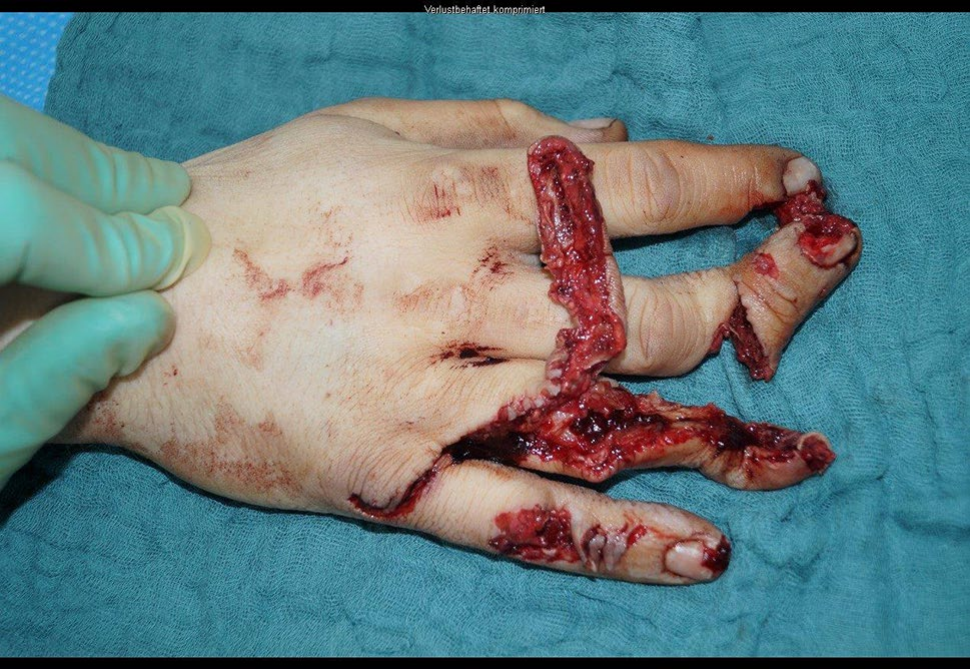
Case Presentation 2: Soft tissue picture immediately after injury

**Fig. 12:**
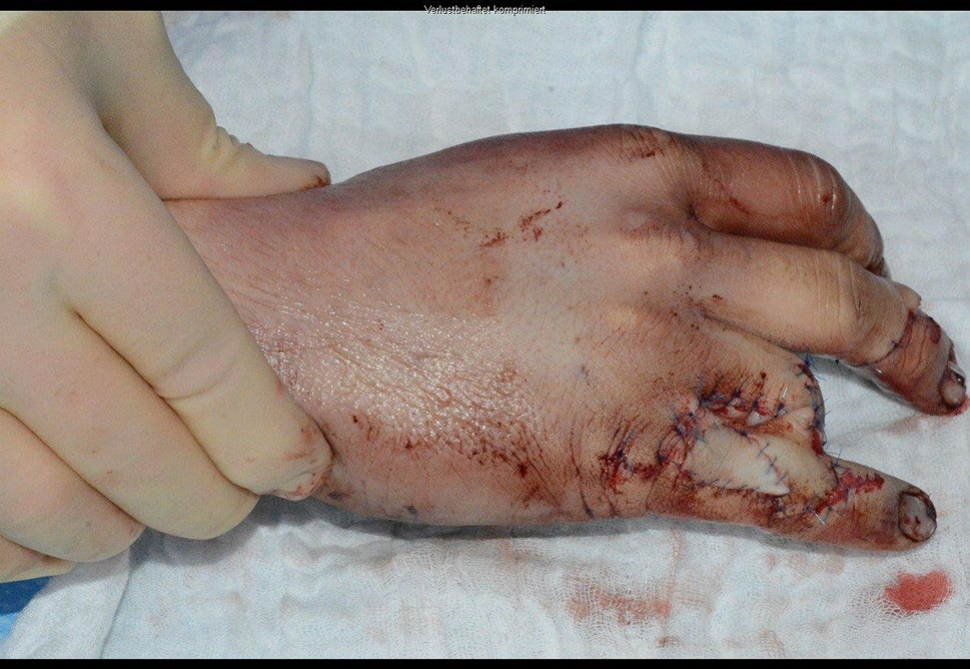
Case Presentation 2: Immediate postoperative result after primary treatment

**Fig. 13:**
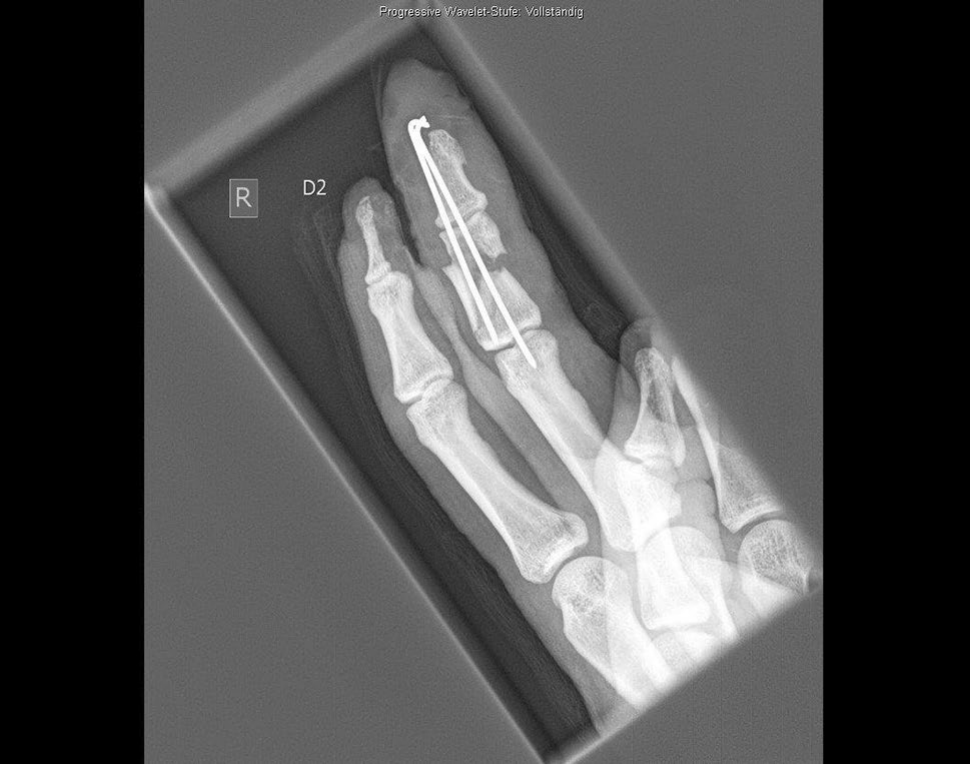
Case Presentation 2: Postoperative X-ray after the first operation

**Fig. 14:**
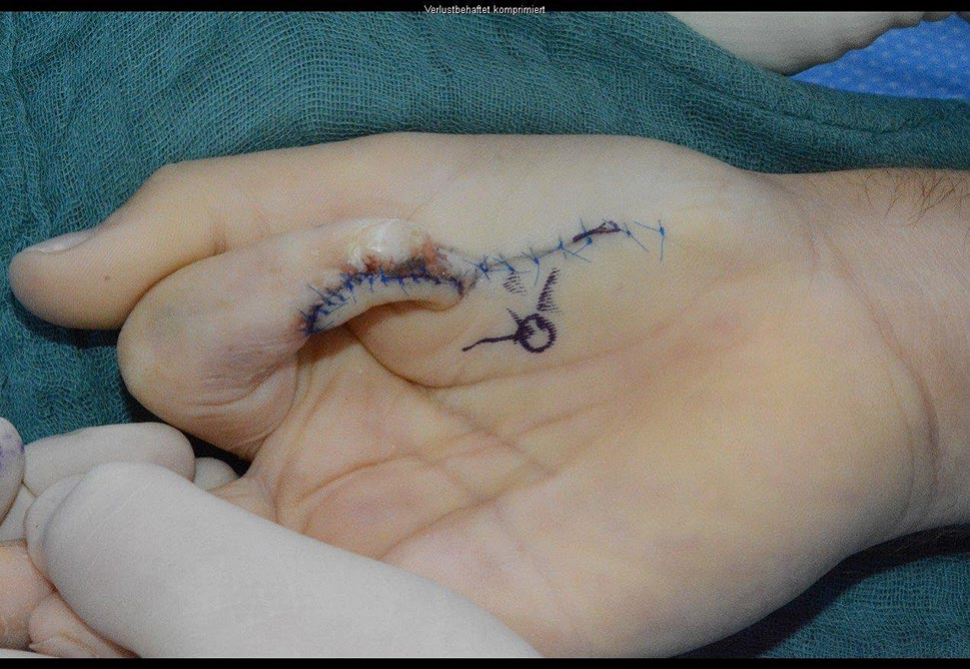
Case Presentation 2: Thenar flap for fingertip coverage

**Fig. 15:**
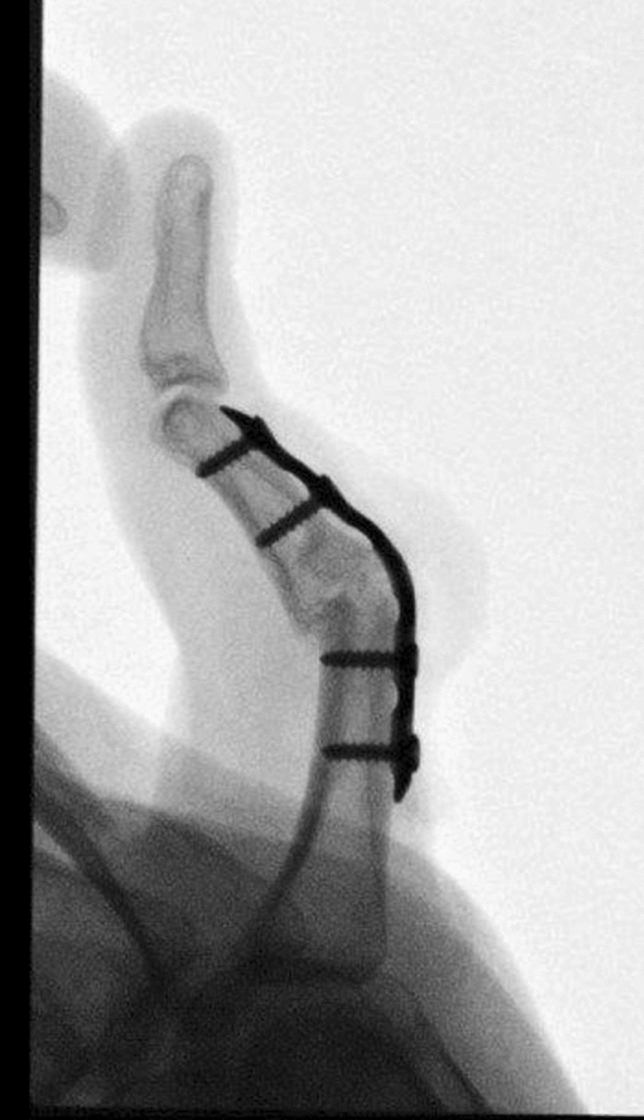
Case Presentation 2: PIP arthrodesis with dorsal plating in cup-and-cone technique

**Fig. 16:**
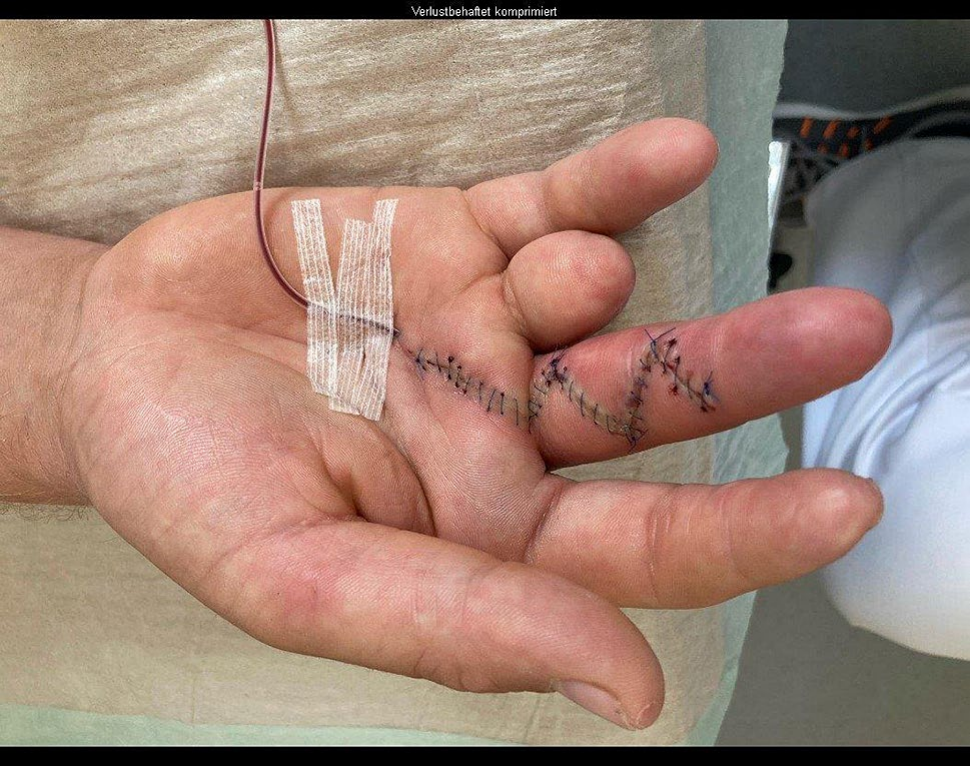
Case Presentation 2: Immediate postoperative result after last operation – mark the pulp of the index finger

A 24-year-old male patient, who tried to work with a circular saw presented with partial amputation of the middle and destruction of the ring finger as well as severe soft tissue loss of the ulnar tip of the index. Primary treatment consisted of k-wire osteosynthesis of the middle finger and semi-occlusive dressing for the index finger as well as full thickness skin graft of the fifth finger. A healing disorder occurred during the course requiring a thenar flap to cover the index fingertip and a secondary PIP arthrodesis of the middle finger. Nowadays he is back at work (Figs. 10, 11, 12, 13, 14, 15, 16).

#### Case 3

**Fig. 17:**
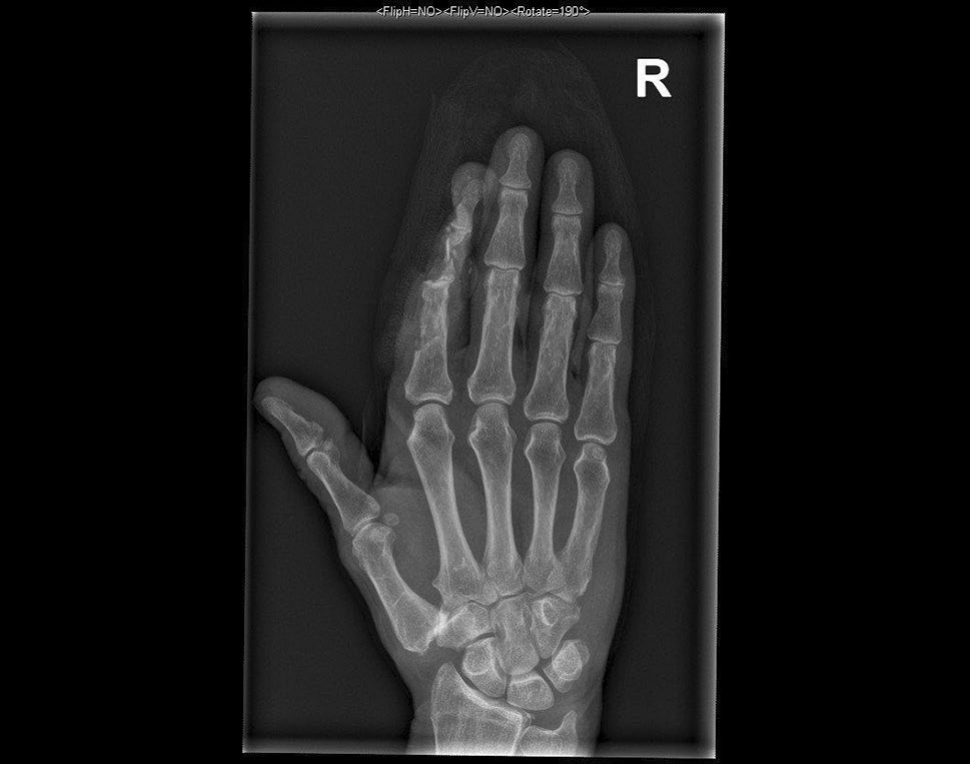
Case Presentation 3: X-ray after circular saw injury with loss of a major part of P2 and P3 of the index finger

**Fig. 18:**
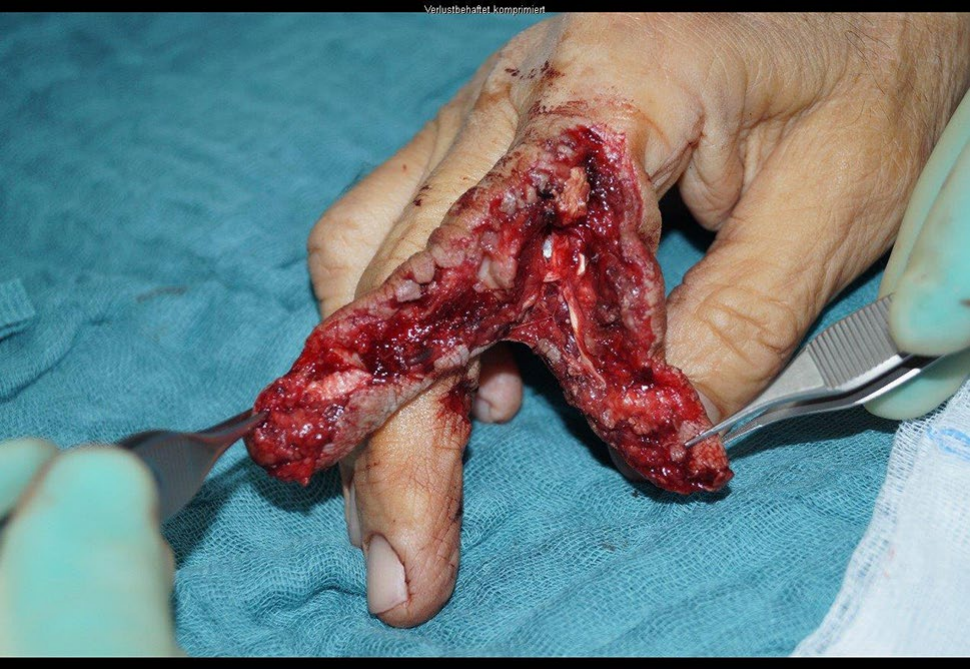
Case Presentation 3: Soft tissue situation immediately after injury of the index finger

**Fig. 19:**
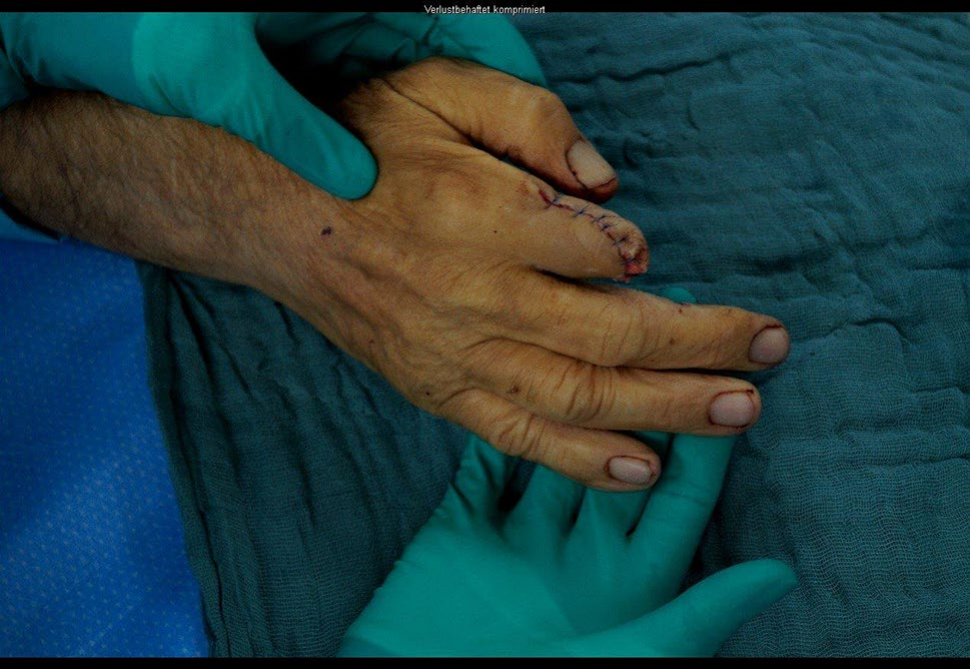
Case Presentation 3: Postoperative clinical picture after stump formation dorsal aspect

**Fig. 20:**
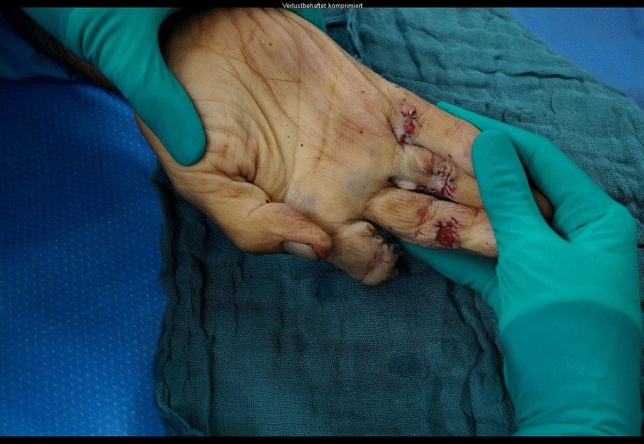
Case Presentation 3: Postoperative clinical picture after stump formation palmar aspect

A 69 year old retired office worker wanted to chop wood with a circular saw at home resulting in a severe damage of the second and third phalanx of his index finger, which could not be restored. He sustained other superficial injuries in the palmar side of the other fingers of his hand. PIP exarticulation and soft tissue management was performed (Figs. 17, 18, 19, 20).

## Discussion

The most important finding of the present study was that, although the incidence of hand injuries presenting at a level 1 trauma center during lockdown period in 2020 decreased, the total number of injury related hand surgeries increased in the same period. Furthermore, a shift from work related trauma mechanisms to DIY and household injuries was found.

Data of a level 1 trauma center with license to treat any kind of hand injury including occupation related severe injuries (BG-SAV) was collected retrospectively. Therefore, all acute injuries were taken into account. In addition, the 60 km catchment area includes rural and urban patient clusters. Thereby a reliable cross section of private and occupational hand trauma representative for German hospitals can be drawn.

The nonsignificant reduction of 18.3% of hand trauma during lockdown is supported by findings of previous studies from other European countries [9–12]. Poggetti et al. investigated isolated hand traumata in Florence, Italy, during the first two months of the Covid period and showed a significantly decreased total number of orthopedic and trauma patients, while hand traumata were only slightly affected [9]. Contrary to the present study, conservative treated patients were excluded. Similar Ho et al. showed no change of hand trauma patients within lockdown period, but a shift of patients towards a higher age [11]. This trend has not been found within our patient collective.

Kreis et al. evaluated the total number of general trauma patients within the equal period and setting in the same department [13]. The total number of emergency patients presenting at the same trauma department during lockdown decreased by 27% in comparison to 2019 [13]. Similarly, the rate of high urgent surgeries was higher during lockdown period. While the number of elective surgeries was significantly reduced in the study of Kreis et al., this has not been in focus of the present study.

Further similarities between hand and general trauma during lockdown can be found in the significant increase of DIY related injuries by simultaneously decreased total trauma number. Additionally, when matching data of Kreis et al. with our data, in 2019 32.3% of trauma emergencies were hand traumata, compared to 36.5% during lockdown period, underlining the necessity of hand surgery in a maximum care hospital—especially in these times.

Regarding trauma mechanism occupational and private injuries were distinguished. While 34% were occupational in 2019, in 2020 only 16.5% of total hand injuries were work related showing a significant decrease of 51.7%. Taking into account, that based on the German insurance system only selected hospitals have the approval to treat severe occupational accidents (called BG-VAV and BG-SAV), occupational injuries might even be over-representative within our collective in both timeframes.

The analysis of exact trauma mechanism revealed a decrease of (occupational) cooking and industrial injuries during lockdown with an increase of DIY injuries caused by electrical saws or gardening. Prevalence of other injury mechanisms, such as sport traumata, traffic accidents or brutality offenses were relatively constant.

Poggetti et al. [9] similarly found enhanced domestic accidents within their patient collective, whereas “traffic-related, sports-related and fortuitous injuries significantly decreased”. However, in the study by Pogetti et al. mechanism of injury, injury pattern and surgical interventions were not taken into account [9].

The prevalence of surgical interventions during lockdown period was investigated in a study from Italy [14]. A significant reduction of both, elective and urgent surgeries (by 92.3 and 37.2%, respectively) was found. Especially reduction in urgent surgeries seems contrary to the present study and further published data [15]. In the present study a significant increase of surgery rate was found—indicating more severe traumata in presenting patients. In contrast to the studies carried out in Italy [9] all injuries to the hand were included, independently of severeness. Due to a decreased number of acutely presenting patients with an increased rate of surgical treatment (60 in 2019 vs. 67 in 2020), we conclude that lockdown might have kept patients with a less severe trauma from referring to the emergency department. The same tendency was found in regard to medical emergencies in Germany [3, 4].

Regas et al. published data from a hospital in France showing similarities to our findings [16]. Within their collective surgical treatment was required in more than 75% of cases—mainly caused by domestic accidents. Nevertheless, the characteristics of the injuries that occurred in 2019 are not shown in detail, so that comparison and evaluation of the data is very difficult.

Data from the present study is of clinical relevance, considering the recurrent lockdown periods in different countries since the beginning of the pandemic. It has been shown that the incidence of hand trauma was affected less by the lockdown period than the incidence of general trauma, based on data from the literature. Furthermore, the rate of necessary surgical treatment in hand trauma was even increased during lockdown period, which might be an important consideration for health care and hospital strategies during future lockdown or pandemic periods.

An important limitation of the present study is the retrospective study design. Although detailed documentation enabled assessment of comparable data for all patients, it was not possible to determine clinical scores like the Hand Injury Severity Score (HISS) in every case. Severity of the injury was determined by the need for surgical treatment. However, clinical follow up examination was not performed.

## Conclusion

Although Covid has negatively affected many other medical fields, lockdown has led to a significant increase of domestic hand injuries as well as an increase of surgical interventions necessary in those patients.
